# Victoria House, Chalfont St. Peter

**Published:** 1898-01-29

**Authors:** 


### VICTORIA HOUSE, CHALFONT ST. PETER.

MR. MAURICE B. ADAMS, F.R.I.B.A., architect of the above, writes: My attention has just been directed to a descriptive article on the Homes at Chalfont in which you refer on December 25th last to the closets to be used by the inmates of this building as having "no windows or through ventilation, such light and air as they receive being obtained from the opposite side of the room, containing the lavatory, in which they are placed." Permit me to say that the writer is quite in error, as these closets are thoroughly well ventilated, having a lantern turret over and ample light and air space, and moreover they are isolated from the building in a complete way. As I have had an exceptional experience in sanitary work, I am fully in agreement with the views you advocate, but I think it only fair that this correction should be made.

